# A Treatise on the Diseases of Infants; Founded on Recent Clinical Observations and Investigations in Pathological Anatomy, Made at the Hospice des Enfans-Trouvés; with a Dissertation on the Viability of the Child

**Published:** 1841-01

**Authors:** 


### ART. XIII.

1. *A Treatise on the Diseases of Infants; founded on recent Clinical Observations and Investigations in Pathological Anatomy, made at the Hospice des Enfans-Trouvés; with a Dissertation on the Viability of the Child.* By C. M. BILLARD, M.D., &c. *With Notes*, by Dr. OLLIVIER, of Angers. Translated from the third French Edition, *with an Appendix*, by JAMES STEWART, M.D.—*London and New-York*, 1839. Large 8vo, pp. 620.

2. *A Practical Treatise on the Management and Diseases of Children.* By R. S. EVANSON, M. D., &c., and HENRY MAUNSELL, M.D., &c. Third edition, revised and enlarged.—*Dublin and London*, 1840. 8vo, pp. 498.

THE emphatical sentence of Morgagni may be applied with just as much propriety at the present day as it was by that great pathologist at the time when he wrote, "How vast and new is the space that is still open before us for the study of the diseases of young children." In this country there are few, or indeed no opportunities upon an extended scale for the exercise of talent and industry in the prosecution of this study. We have no public hospitals expressly for young children, and many causes conspire, especially the prejudices of parents and the meddling interference of nurses, to render any practical knowledge of the diseases of infants that can be derived from even the most extensive private practice, not a little uncertain to the practitioner himself, and but little profitable to science, in comparison with that which may be obtained in a public institution where we can be assured our treatment is not interfered with. "They manage these things better in France;" and it is perhaps only in Paris that the diseases of children may be studied to the greatest advantage. In 1828 the number of beds at the Hôpital des Enfans was nearly 600, and we believe it is now considerably increased. The establishment is appropriated to the treatment of sick children, from two to fifteen years of age, of either sex, and whatever may be the disease under which they labour. In this admirable school for practical instruction, M. Billard held for some time a very important station, and there he laboured with almost unrivalled industry and talent to shed new light upon the general pathology and treatment of infantile diseases. The principal object of his work is to exhibit the peculiar characters of these, and to consider them in relation to the alterations which the organs have undergone. He passes each system successively in review, and describes the varieties of form and appearance of every organ, with reference to its healthy, abnormal, and pathological condition; carefully estimates the value and importance of the leading symptoms of disease, and then points out the method of treatment. The development of organs is briefly discussed, and only those congenital malformations are noticed which more or less disturb the function of organs. Neither fevers nor intestinal worms are treated of, because they are of rare occurrence in newborn and sucking infants: to which class the author confines his attention. The very general absence of all febrile reaction in young infants, when at the same time there exists various serious lesions, and the readiness, on the contrary, with which fever is excited by the slightest cause in those who are teething, impress on these two periods an important difference in the character of their diseases.

The work of Drs. Evanson and Maunsell, the first edition of which we noticed in our Third Volume, is an excellent compendium of the best practice of the day on the management and diseases of children; and, as a book of reference and instruction for students and young practitioners, we think it likely to be more useful than that of M. Billard, which must be regarded rather as a valuable pathological record than as a ready guide in the moment of doubt and difficulty. M. Billard's work, too, is almost exclusively confined to the consideration of infantile disease, while Drs. Evanson and Maunsell take the wider range of the whole period of childhood.

Before he enters upon the study of particular affections, M. Billard considers, in the first part of the work, the general phenomena which are presented upon examining the external condition of the child. The second part comprises the history of diseases developed both during intrauterine life and after the period of birth.

It must be evident, although the fact is too frequently lost sight of, that a precise knowledge of the phenomena which are exhibited upon the examination of the child, and which it is necessary to consider in all diseases, such as the expressions of face, crying, circulation, &c., is indispensable. If we are familiar with these in a state of health, it will, of course, be much more easy for us to appreciate the modifications they undergo in disease.

*The colour of the skin* of new-born children equally deserves attention. Infants recently born are almost always of the same colour. Blood predominates in their tissues, and communicates to them its hue, and the face, body, and limbs are all strongly coloured. From the fifth to the eighth day after birth this hue diminishes, but sometimes continues longer. This red colour is purely accidental, and upon its disappearance is followed by other hues of various character. If it continues, it is not so intense as at first; it becomes of a violet hue, particularly in the hands and feet. The alteration, however, is not always an evidence of health, for it often coexists with an œdematous swelling of the limbs. If the finger be applied to the skin of an infant the red colour disappears at this point, and it becomes yellowish : afterwards the blood returns by degrees in the capillaries from which the pressure had removed it, and the yellow tint is replaced by the previous red. Very often we observe, after the red colour has disappeared, that the skin exhibits a universal tint of yellow, and sometimes of a copper colour. By physicians in general, this appearance of the skin is erroneously thought to indicate an affection of the liver.

The phenomena which precede, accompany, and follow the separation of the funis are described in a very novel and instructive manner. The desiccation of the cord and the time of its separation from the abdomen differ much in different infants. The desiccation of the cord is altogether a physiological phenomenon. That part of the cord attached to the placenta does not dry like the portion attached to the abdomen of the child, but shrinks and decays like a dead substance, whilst the abdominal portion is not so affected. Here the desiccation ceases as soon as life is extinct : it either does not proceed in still-born children, or it is considerably retarded. Instead of dying and separating at the end of a few days, as is observed during life, the cord undergoes in the dead body a perfect decomposition, differing entirely from its normal desiccation. This fact, which it may be difficult to explain, should be borne in mind, as it is important in reference to legal medicine. If a fœtus be examined some time after birth, if the cord still remain attached, we ought to examine whether it exhibits the characters of a normal desiccation, or whether it is soft or in a state of putrefaction, like the general condition of the dead body ; for, in the former case, the child could not have been still-born, but might have lived one or two days, since the desiccation, which only exists during life, had already commenced, while in the latter the infant was still-born, or had lived but a short time. Such is the importance of this fact, that M. Billard particularly calls attention to it, as it may, in conjunction with other circumstances, prove whether the child was born alive ; as the principle can be laid down, that in every instance in which the cord is dried, flattened, twisted, and blackened upon the dead body of an infant, it has lived—at least one day ; this condition never being produced on a dead body. It appears that the ordinary time at which the cord separates from the infant is the fourth or fifth day ; but, as this rule is subject to many exceptions, it is not entitled to much confidence in a legal point of view, when we wish to determine the precise age of the infant.

*Exfoliation of the epidermis* does not occur until after birth. M. Billard knows no case in which it ever commenced before ; and it is worthy of remark, that premature children never exhibit this phenomenon : some time must elapse, and the infant arrive at a certain age, before it occurs. Authors on legal medicine have endeavoured to draw certain inferences from the separation of the epidermis in relation to the age of the infant. Orfila, desirous of investigating the statements of Chaussier, Capuron, &c. upon this subject, made considerable researches with M. Thierry, and he concludes that the epidermic exfoliation exhibits at first a preparatory stage, next an elevation of the epidermis, and, lastly, its separation; and that the preparatory stage can be observed from the sixth to the eleventh day, the elevation of the epidermis on all parts of the body from the twentieth to the thirtieth day, and the complete exfoliation from the thirty-fifth to the fortieth day: these authors also state that certain diseases retard or suspend this process. M. Billard has not been able clearly to observe the preparatory stage mentioned by Orfila and Thierry. He found that, in eighty-six infants, the commencement of the exfoliation and its duration were very variable, and that from three to five days appears to be the age at which the epidermic exfoliation is at its greatest height. In forty-two of these infants it was not observed at all: it not unfrequently occurs insensibly; the epidermis comes off in a dust, and the different periods of exfoliation cannot be detected. The cause of the epidermic exfoliation in new-born children is susceptible of a satisfactory explanation. The epidermis, until the period of birth, is soaked in the liquor amnii; when exposed to the air it becomes suddenly dried, and loses the suppleness maintained by the fluid medium in which it is immersed during intra-uterine existence: hence results a cracking and scaling of the epidermis, and its final separation in the form of scales or powder. It is difficult to establish any constant conformity between the separation of the epidermis or that of the funis, and the age of the infant: even the attempt to draw any general inferences from these phenomena has been fruitless. It is certain, however, that the epidermic exfoliation of young infants is a natural and healthy phenomenon.

In a very young infant, the means of expression are limited to the cry and appearance of the face; and both require attention, that we may be enabled to distinguish a state of health from one of threatened or really existing disease. M. Billard enters minutely into these subjects, and throws out many hints that well deserve the notice of practitioners. It may be worth while to observe that very young children rarely shed tears when they cry: the secretion of the lacrymal gland is excited, as is well known, immediately and sympathetically by sorrow; but are children of a very tender age under the influence of mental emotions? Is this secretion produced by any other influence than the nervous excitement proceeding from some moral cause; and are physical distresses, which appear to be the only kind endured by a being whose brain cannot as yet combine ideas, and from which there appears to emanate no volition, capable of acting on this gland? It is difficult to answer these questions. The lacyrmal gland at this period is perfectly developed; it receives arteries and nerves, and anatomically resembles other glands, but no tears flow while the young infant cries from sickness or pain. This fact is physiologically curious, and is a remarkable example of the particular influence of the nervous system on the functions of certain organs of the body. We must refer to the work itself for M. Billard's "analysis" of the distinctions between the infantile cry of health and pain or disease.

Next to the cry, the expression of the face is one of the principal means by which the child manifests the sensations it experiences. In order to add to our knowledge of the symptoms of disease in children, M. Jadelot has proposed a *physiognomical semeiology*, which no doubt assists our diagnosis of the diseases of infants. M. Billard gives a slight sketch of M. Jadelot's doctrine, and describes minutely and accurately the different expressions of the infantile countenance with which the physician should be acquainted. Of this system of signs, the reader will find some account in our Second Volume, p. 356.

After a few brief remarks on the "state of the pulse in children and feebleness at birth," we come to the second part of the work. The first chapter, on diseases of the skin, contains no important addition to our previous knowledge of the subject, with the exception of the section on "Œdema, or induration of the cellular tissue of new-born children." This hard or indurated state is manifested by a swelling of the limbs or face, which are more or less coloured, and give firm resistance to the touch; the sensation, therefore, produced by touching gave rise to the above term. Anatomical examinations, however, have proved the vagueness of such expressions, and the denomination compact œdema has been proposed instead of induration. This title is more correct, inasmuch as in this disease there is no induration of the cellular tissue: it feels hard because it is distended with fluid, and undergoes no other change than that which results from mechanical distension. There always has been, and there still exists much difference of opinion, even among the best authorities, as to the nature of this disease. M. Billard's opinions are entitled to attention, as he has had numerous opportunities of investigating every circumstance connected with its origin and progress. He believes that the following truths have been established: 1st. The induration of the cellular tissue in young infants is simple œdema, analogous to the œdema of adults: it may be either local or general, and ought always to be distinguished from induration of the adipose tissue. 2d. It is more common in winter than in summer, and more frequent in young infants than in those of more advanced age; the predisposing causes are the natural feebleness of the child, and a state of general and congenital plethora: a superabundance of venous blood in the tissues, and a dry state of the skin before the exfoliation of the epidermis.—The immediate causes are, 1st, an obstruction in the course of the blood, resulting from its quantity in the blood-vessels; 2d, its engorgement in the cellular tissue, to which it furnishes too much materials for secretion; 3d, the action of external agents on the skin, which, without condensing the venous blood, as has been asserted, are yet capable of suspending the cutaneous transpiration, and thus favour the accumulation of serum in the cellular tissue: the sanguineous engorgement of the liver, lungs, and heart, the persistence or closure of the fetal openings, are not the exclusive and indispensable causes of this affection. 3d. When œdema is general, and the venous congestion is carried to a high degree, all parts, where there exists cellular tissue, undergo a disturbance in the functions which they discharge. Thus the glottis becoming œdematous at the same time that the lungs are the seat of congestion, the cry of the child is generally painful, acute, and smothered. The slowness of the circulation easily accounts for the coldness of the limbs, and the great debility; and thus may be explained all the symptoms described by authors. 4th. The therapeutic indications are, 1, to relieve, by suitable evacuations, the general plethora; 2, to excite the skin by irritating frictions, by the use of woollen garments next to the skin, and the adoption of all means proper to establish cutaneous transpiration. Vapour-baths are less useful than frictions and woollen to the skin: Billard has often known these succeed. The respiration of a child during its continuance in the vapour-bath is painfully accelerated, and congestion or effusion on the lungs or brain has been known to follow its employment. Dr. Maunsell observes (p. 184), that no febrile condition accompanies this affection; wherein it differs from infantile erysipelas, which, in other respects, it much resembles. The child will not suck, is restless, and continually whines. As to the treatment, he states correctly that the earlier plans had for their object the application of heat by means of the warm and vapour bath, and wrapping the child in wool or cotton. "The lowness of temperature, however, is merely a symptom of the state of commencing asphyxia, and that state is not likely to be removed by the application of external heat alone. We must endeavour to lessen the venous congestion, and, if we succeed in doing so, the production of heat will proceed naturally. For this purpose we would recommend friction with warm flannel; the administration of an emetic of ipecacuan, for the purpose of removing mucus, and exciting respiration, and the internal use of stimulants, as warm wine-whey. We cannot put forward this plan as the result of experience, but we conceive it to be based upon a rational view of the pathology of the disease. Paletta recommends leeches to be applied to the œdematous parts, as a means of promoting circulation; but his plan has not suceeded in other hands." (Maunsell, p. 185.) Dr. Carswell makes some interesting remarks upon this disease in his article "Induration," in the Cyclopædia of Practical Medicine. One fact is worthy of notice. The great mortality of the Hospice des Enfans has been attributed to induration of the cellular tissue; this, M. Billard believes, is incorrect. There often exist at the same time affections of the brain, lungs, and intestines, much more serious than œdema, and much more fatal. Fifty children died in 1826 of œdema, or induration of the cellular tissue, without any serious lesions of other organs. In May and November the greatest number affected with induration were admitted, and all these patients died of affections of some important organ, and more frequently of the lungs. When œdema is local, or if it be general and yet not severe, it is not to be regarded as a fatal disease, nor will it become so until it is complicated with disease of some vital organ. Of all the phenomena accompanying œdema of infants, icterus is one of the most common. In seventy-seven infants affected with œdema, thirty were jaundiced, but no organic lesion was detected that could account for this difference. The whole subject of exanthematous diseases is more practically, and therefore more usefully considered by Dr. Maunsell than by M. Billard. Dr. Maunsell very properly corrects one statement of M. Billard with respect to the eruption of measles. "Billard describes the eruption of measles as not feeling elevated above the surface, which is decidedly contrary to the fact." (Maunsell, p. 382.)

The chapter on the diseases of the digestive apparatus contains much valuable information, especially as to the pathology of the various maladies that are described. We shall confine our notice to the section on gangrene of the mouth, in preference to giving a mere sketch of each division of this elaborate and instructive chapter. Gangrene of the mucous membrane of the mouth may occur in infants in various ways. It is not an unfrequent termination of aphthæ. When aphthæ become gangrenous, their edges shrink and assume a burnt, torn, and flabby aspect; then a brown eschar often forms in the centre, which soon detaches itself, leaving a granulated surface of a vermilion colour. In place of an eschar, the centre of the ulcer sometimes gives off a creamy substance of a brown colour, and of a gangrenous odour. The surrounding parts tumefy, assume a violet aspect, become softer, and are easily depressed. A ropy saliva flows from the child's mouth. The face becomes pale, the patient remains drowsy, and sinks without having exhibited any febrile reaction or cerebral excitement. The pulse always remains very feeble, and the skin is remarkable for its paleness and insensibility. To these symptoms are often added vomiting, diarrhœa, distension of the abdomen, and sometimes hiccup and frequent eructations. This termination of aphthæ is extremely fatal, for it happens usually at a period when the child, wasted by the previous phlegmasia, affords no opportunity for appropriate treatment. As soon as the gangrene has formed it should be touched with slightly acidulated gum-water. Should this application effect no alteration in the aspect of the ulcer, sulphuric or muriatic acid must be used. In order to apply them in the easiest manner, a glass capillary tube may be used, immersing one end in the acid, and drawing up one or two drops, which are to be dropt on the surface of the ulcer with the end of the tube. After the application of these acids, and when the eschar is detached, the remaining gangrenous part must be touched with solid nitrate of silver sharpened at the point: for by using the acid again, it might touch the parts deprived of the eschar, which are in a state of extreme irritability. It is much easier to moderate and limit at will the action of nitrate of silver. These practical remarks will apply to all ulcers of the mouth which assume a gangrenous character. There is another kind of gangrene of the mouth, which does not follow any well-marked inflammation, but which appears to be caused by a particular alteration of the parietes of the mouth. This disease has long attracted the attention of the profession, but it is but recently that we have had any very satisfactory account of it in the works of Baron,* Guersent, Jadelot, and Isnard;† M. Billard gives the detailed account of several examples of it which occurred at the hospital. There are usually two well-marked stages of this disease: 1st. An œdematous, circumscribed tumefaction, characterized by an oily aspect of the skin, and by a central body more or less hard, on which there is sometimes an obscure red spot, either on the internal or external surface of the cheek: this is the first stage, and in young infants is not accompanied by fever or any symptom of reaction. 2d. This central part presents an eschar which usually forms from within, the mucous membrane becomes disorganized, the bones are laid bare, all the soft parts, even to the periosteum mortify and separate in shreds, at the same time that the mucous or bloody matter, mixed with the remains of the gums or sides of the mouth, flows out, exhaling a disgusting odour: this is the second stage. Gangrene of the mouth must not be confounded with malignant pustule, for, as Rayer has well observed, the gangrenous inflammation commences in the interior of the mouth, and from thence spreads to the skin. It is not proved to be contagious, and it is usually observed in one patient at a

* Mèm. sur une Affection gangreneuse de la Bouche. Bulletin de la Faculté.

† Diss. sur une Affection gangreneuse particulière aux Enfans. Paris, 1818.

time in an hospital, even when there are many other children in the same ward. It appears difficult to explain the cause of this gangrene; the fact should be borne in mind that œdema and indolent tumefaction always precede the formation of the eschar. M. Billard thinks it does not proceed from inflammatory action, but that it is the result of an indolent engorgement, analogous to that which constitutes anasarca.

M. Billard attaches but little importance to any general treatment for this severe disease; the slowness and uncertainty of its action will never compensate for the advantages of caustic applied directly to the seat of a disease, the progress of which is of so frightful a nature. The strength of the child should always be supported by nourishing diet, as equal parts of milk and broth, and "a few teaspoonsful of Malaga wine in the course of the day." Internal stimulants, however, must be cautiously employed, for notwithstanding the apparent feebleness of the child, the intestinal canal is not unfrequently the seat of great irritation, or even inflammation. Dr. Evanson, treating of the same disease in children of a more advanced age, recommends (p. 222), in addition to the early application of muriatic acid, as the only efficient local application, the administration of bark, wine, and the mineral acids, with a nutritious diet.

It would be useless for us merely to enumerate the various maladies of the digestive apparatus of infants which M. Billard describes; but in justice to him we must say that he has given an admirable account of the whole subject, and that his opinions are instructively illustrated by the detail of the most striking cases that came under his notice.

Under the head of diseases of the respiratory apparatus, an equally minute account is given of the principal affections of the nasal fossæ, the larynx, trachea, and lungs. There is one condition of the larynx and trachea, which, without any lesion whatever of the mucous membrane, especially merits the attention of accoucheurs. We allude to the abundant secretion of mucus which in some infants obstructs these passages so as materially to impede the establishment of respiration. This affection is usually accompanied by a peculiar alteration of the cry, which is husky and incomplete. "It is probable," M. Billard says—we should say certain, "that this mucus is accumulated in the larynx and trachea before birth." The effects of this accumulation are generally trifling, and of short duration; sometimes, however, at the time of birth the respiration of the child is thus seriously impeded; and if the practitioner is ignorant of the fact, or careless respecting it, a state approaching to asphyxia may arise, which may be prevented by removing the abundant mucus by a feather introduced into the entrance of the larynx, where it usually adheres. M. Billard's observations upon croup are brief but interesting. Croup, it is well known, consists of an inflammation of the larynx and trachea, complicated with the rapid formation of a pellicular concretion spread over the walls of the larynx, which extends in some cases to the trachea and bronchi. The remote causes appear to be the same as those of laryngitis or bronchial catarrh; but it is difficult to explain in a satisfactory manner the immediate cause of the formation of the false membrane which occurs in this affection.* It

* We would especially recommend young practitioners to refer to Valentin's Recherches historiques et pratiques sur le Croup.—Rev.

is almost always during the prevalence of epidemic catarrh, or hooping-cough, that the croup is most rife; it precedes or accompanies one or the other of these phlegmasiæ, and is sometimes even a complication of them. M. Bretonneau has in vain attempted to separate the connexion existing between the catarrhal affections and croup, and to controvert the opinions that have been held for half a century by Home, Rosen, Michaelis, and supported by Jurine, Double, Vieusseux, Royer Collard, Blaud, Valentin, Bricheteau, and Desruelles.* A few writers, adopting M. Bretonneau's views, have endeavoured to prove with him that there is something specific in the nature of croup; but without admitting this, the formation of the false membrane may, M. Billard thinks, be explained to a certain extent. He has discussed this subject in detail in another work,† and the following are, in brief, the reasons he adduces to show in what the peculiar nature of croup consists. 1st. There exists, as it were, but a degree between the thick, tenacious, filamentous mucus with which inflamed mucous membranes are covered, and the membranous exudation of croup. 2dly. The membrane of croup presents nearly the same chemical elements as this mucosity where fibrine predominates. The same analogy exists between the pellicular excretion of muguet and the mucosity of catarrhal affections; so that the puriform mucosity of catarrh, the false membrane of croup, and the excretion of muguet appear to be but alterations of the same secretion, and vary only in form, and the parts they occupy. 3dly. Before this membrane appears, the mucous membrane is always much inflamed, red, and gorged with blood; the subjacent tissue participates in this injection, and when the inflamed membrane is at the same time the seat of sanguineous exhalation, this exhalation is accompanied or followed by pellicular concretions, from which it is to be inferred that croup is a catarrhal phlegmasia, but that the blood destined to the secretion of mucus is, in the case under consideration, concentrated in greater abundance, or rendered plastic by inflammation, and imparts to the mucosity that part of its composition which concretes the quickest, that is, the fibrine; whence arise the striæ, pellicles, and white patches with which the mucous membranes, affected with muguet or croup, are covered. Children at the breast are much less subject to croup than those of a more advanced age; it is most prevalent from two to ten years of age. Young infants however are liable to pellicular inflammations of other mucous membranes, such as those of the mouth, œsophagus, and nasal fossæ, whilst the opposite condition exists in children of more mature age. Age, therefore, and the organic modifications which belong to it, and which can more easily be understood from their effects than by their physical appearances, seem to produce a difference which ought to be noted, though we are unable to explain it. "But, on the other hand, the readiness with which symptoms of suffocation arise when the slightest inflammation arises in the air-passages of young infants, renders the ordinary tracheal and laryngeal affections almost as dangerous as croup." We have seen many cases which proved the soundness of this statement, and which showed how careful and guarded should be our prognosis of,

* Traité Théorique et Pratique du Croup. Paris, 1824.

† Archives Générales, &c. December, 1826.

and how narrowly we should watch every attack of laryngo-tracheal inflammation in infants. We do not stop to notice the rather unsatisfactory description M. Billard gives of the symptoms and treatment of croup. He "pushes" calomel farther than most of his brethren, for he "never employed calomel except in doses of 18 or 20 grains in twenty-four hours." He always conjoins with calomel direct antiphlogistic means, as leeches to the larynx and trachea, and emollient drinks and topical applications. The practice advised by Bretonneau is, in our opinion, very wisely deprecated, of opening the trachea and introducing calomel or alum to destroy and remove the membranous pellicle. In one case M. Billard did introduce a tube into the larynx covered with a solution of alum, and the child died convulsed in five minutes.

M. Billard observes that death almost always suddenly terminates this frightful disease, against which the resources of art are too often powerless. Dr. Maunsell (p. 324) wisely abstains from so very fearful a prognosis. He admits that croup is always a dangerous disease even under the most favorable circumstances; but it is also one which admits of the use of decisive means, and is thereby remarkably within the control of art. When we see a patient then at an early period of the disease we may hope to relieve him. Upon the much-disputed point too of spasmodic croup as a distinct disease, Dr. Maunsell's observations exactly agree with our repeated experience:

"Every affection of the larynx is subject to exacerbations, which partake much of a spasmodic character; and a paroxysm of this nature may occur at an early period of true croup, and destroy the patient before there has been any time for any very important results of inflammation to be produced. Similar paroxysms have also been observed without previous or subsequent symptoms of inflammation, and have subsided without active treatment, so giving rise to the notion, that there existed no distinct form of spasmodic croup. There are, however, no means of distinguishing between the two affections, (if two distinct affections exist,) beyond the degree of violence of the symptoms. Whenever, therefore, we meet with the symptoms already enumerated, as indicating the onset of croup, we should be upon the alert; and as soon as any permanent difficulty of breathing sets in, we should forget all hypothesis of the spasmodic nature of the disease, and treat it as an active inflammation, persuading ourselves, like Dr. Kellie,* that there is truly no essential difference between them (spasmodic and inflammatory croup) other than what arises from degrees of violence and the obvious circumstance of intermission and continuance." (Maunsell, p. 324.)

From frequent experience at the Hospice des Enfans, M. Billard infers that pneumonia of infants exhibits peculiar characters, in which it differs from the same disease in adults. Instead of being an idiopathic affection, arising from irritation developed in the pulmonary tissue, the pneumonia of young infants is evidently the result of a stagnation of blood in their lungs. Under these circumstances the blood may be considered as a foreign body, and it concurs in producing an alteration in the pulmonary tissue with which it combines, and is identified with it so as to form hepatization of the lungs. It appears, therefore, that inflammation of the lungs, which produces hepatization, arises in infants, in general, from some mechanical or physical cause, which is not the case in adults; besides, the inflammation is usually very circumscribed, and is almost always found limited to a point primarily engorged; and the pleura, which

* Letter in Cheyne's Pathology of the Larynx and Bronchia.

generally is inflamed with the lungs at a more advanced age, is not affected in young infants. The inflammation once developed, as in the adult, may give rise to various alterations of tissue, from simple hepatization to a great disorganization of parts. The details of several cases are given to illustrate these opinions.*

In the chapter on diseases of the "circulatory apparatus," M. Billard gives the result of his researches on the establishment of the independent circulation, which differs in many respects from the account usually given by other writers. He considers in succession the period at which the fœtal openings are obliterated, their mode of obliteration, and the physiological and pathological consequences which arise from these changes. In a number of children five days old the fœtal openings remained open, and none of these children exhibited any peculiar symptoms which appeared to have their seat in the circulatory apparatus. On the eighth day the fœtal openings are usually obliterated, but even at this period they are sometimes found open. Even on the twelfth and fifteenth days the foramen ovale or ductus arteriosus may still be open without the existence of any particular symptom. It is evident, then, that the fœtal openings are not obliterated immediately after birth, and that the period at which the obliteration occurs is variable. The changes which take place after birth in the vascular system occur in the following order: the umbilical arteries are first obliterated, then the umbilical vein, next the ductus arteriosus; and, lastly, the foramen ovale. The existence then of the fœtal openings for some days after birth ought not to be considered as a disease, since it is not uncommon to meet with it without any symptoms of disease at all. We can verify these statements of M. Billard by several dissections we have made. Cyanosis, or the "blue diseases of infants," is by no means the constant result of the persistence of the foramen ovale, nor of the passage of venous blood into the arterial system; there are many examples of malformation of the circulatory apparatus existing, of which we have ourselves two specimens, which might have produced the disease without its ever having appeared. Cyanosis may exist with or without a malformation of the heart, provided the blood, in passing through the lungs, does not undergo the vital and chemical modifications which ought to occur. If, notwithstanding the communication between the two auricles, cyanosis does not take place, it is because the blood passing through the lungs is in sufficient quantity, and sufficiently oxygenated to impart its oxygenation to the venous blood with which it is mixed. On the other hand, if the cavities of the heart are in a normal state, but the peculiar disposition of the lungs does not permit the oxygen of the air to transform the venous into arterial blood, cyanosis will be the result. Local or general cyanosis, however, in new-born children, is in most instances the effect of a sanguineous congestion about the heart and lungs, and the best method of relieving it is that recommended by Corvisart, to hold the child near the fire, and to rub the head and body gently with hot cloths. This may require to be persevered in for some time.

In the chapter on diseases of the cerebro-spinal apparatus, M. Billard treats on congestions and softening of the brain, and inflammation of the brain and spinal cord, of each of which maladies he adduces instructive cases. The description he gives of the progress of purulent ophthalmia in infants, and the destructive consequences that too frequently arise in

* See M. Valleix's observations on this affection, B. and F. Med. Rev. Vol. VII. p. 77.

the structure of the eye from carelessness in parents, or negligent treatment of the practitioner, is not merely more minute, but certainly more instructive than Dr. Maunsell's account of the same serious disease. The treatment is, in our opinion, much too briefly dismissed by both authors. M. Billard contents himself with giving a brief extract from Mr. Lawrence's work; and Dr. Maunsell's remarks upon the treatment are not, we think, sufficiently precise for the information of students and young practitioners upon a subject, which, if not well understood, involves the dreadful calamity of loss of sight. In the general principle of treatment recommended in both works we perfectly agree. Bleeding by leeches, we quite coincide with Dr. Maunsell in thinking very rarely necessary; or the early use of astringent or stimulant collyria in preference to warm emollient applications. But the practitioner will very imperfectly perform his duty if he merely prescribes the proper application. He must use it himself at first, and carefully instruct the nurse how she is to employ it. Upon this very important point nothing is said in either of the works before us; and from want of due attention to it, we have known in more than one instance complete destruction of the eyes to follow, which we have no doubt might have been averted if due caution had been exercised. Again, too, we find no recommendation for the application of the red precipitate ointment to the edges of the eyelids at night, for the purpose of preventing their adhesion, which we think essentially necessary. In our former notice of Drs. Evanson's and Maunsell's work,* we gave it as our opinion that the latter gentleman "underrated the value of chalybeate medicines" in the treatment of scrofula. In the present edition an interesting note is added at p. 468, which seems to confirm our opinion. In a letter lately addressed to the Royal Academy of Medicine, of Paris, by M. Coster, the virtues of iron, as a preventive of the development of scrofula, are highly extolled. Two years ago M. C. placed a number of dogs, rabbits, &c., in the circumstances most favorable to the development of the scrofulous diathesis. Thus many of the unfortunate animals were shut up in dungeons, without light, incapable of moving, and exposed to a moist cold by means of wet sponges which were hung up in the cages. Some of the animals placed in these conditions were fed on their ordinary diet, others were fed with ferruginous bread, containing half an ounce of carbonate of iron to the pound. All the former became ill, the greater part tuberculous, but not one of those fed on bread containing iron presented a trace of tubercles.†

A medico-legal dissertation on viability with reference to the pathology of new-born children terminates M. Billard's work. The Appendix by Dr. Stewart contains many interesting illustrations of and additions to the opinions of M. Billard upon various subjects of practical importance.

This work is decidedly a very valuable contribution to our knowledge of the diseases of infants. No other writer, foreign or English, had better opportunities of studying the pathology of infantile disease than the much-lamented and highly-esteemed Billard; and with strict justice we may add, that none could have turned them to better account. The translator has performed his task in a very correct and creditable manner, and his Appendix forms a very important addition to the original work.

* British and For. Med. Rev., vol. III. p. 446. † Bull. de l'Acad., Jan. 31, 1840.

We have before expressed the high opinion we had formed of the great ability of Drs. Evanson's and Maunsell's much required work; and we need only add, that the present edition is still more deserving of our commendation. It would be unjust to these gentlemen not to observe that their work, although necessarily and properly containing much valuable matter from preceding writers, affords abundant proof of their personal experience and practical judgment.

---

